# Evaluation of peak alignment performance of commercial and open-source tools for GC×GC–MS based non-targeted screening

**DOI:** 10.1093/bib/bbag458

**Published:** 2026-09-02

**Authors:** Andriy Rebryk, Denice van Herwerden, Saer Samanipour, Peter Haglund

**Affiliations:** Department of Chemistry, Chemical Biological Centre (KBC), Umeå University, Linnaeus väg 6, 901 87 Umeå, Sweden; Amsterdam Institute for Life and Environment (A-LIFE), Faculty of Science, Vrije Universiteit Amsterdam, De Boelelaan 1108, 1081 HZ Amsterdam, The Netherlands; Van ’t Hoff Institute for Molecular Sciences (HIMS), University of Amsterdam, 1090 GD Amsterdam, The Netherlands; Van ’t Hoff Institute for Molecular Sciences (HIMS), University of Amsterdam, 1090 GD Amsterdam, The Netherlands; UvA Data Science Center, University of Amsterdam, 1090 GD Amsterdam, The Netherlands; Queensland Alliance for Environmental Health Sciences (QAEHS), The University of Queensland, Cornwall Street, Woolloongabba 4102, QLD, Australia; Department of Chemistry, Chemical Biological Centre (KBC), Umeå University, Linnaeus väg 6, 901 87 Umeå, Sweden

**Keywords:** GC × GC–MS, inter-sample peak alignment, open-source software, Julia programming language, non-target screening, house dust

## Abstract

Comprehensive two-dimensional gas chromatography–mass spectrometry (GC × GC–MS) is a powerful tool for analysing complex mixtures, but its wide use is limited by the lack of robust and transparent inter-sample peak alignment workflows. Commercial solutions often rely on proprietary algorithms, while user-friendly open-source alternatives remain scarce. Here, we introduce jAligner4GCxGC, a new open-access Julia package for inter-sample GC × GC–MS peak alignment and benchmark its performance within practical GC × GC–MS data processing workflows against Guineu (open-source) and ChromaTOF Sync 2D (commercial). The package performs data preprocessing, alignment, and postprocessing including feature merging, library searching, and compound data retrieval. Three indoor dust samples, including certified reference material, were spiked with >150 reference compounds at concentrations of 0.5, 5.0, and 50 pg/μl and analysed using workflows optimized for each application. Performance was evaluated based on recovery of correctly aligned spiked compounds and workflow processing time. Because the software packages differ in peak detection and preprocessing, comparisons involving Sync 2D should be interpreted at workflow rather than alignment algorithm level. At 50 pg/μl, Guineu and jAligner4GCxGC detected 94% and 92% of compounds, respectively, outperforming Sync 2D (82%). At 5 and 0.5 pg/μl, Sync 2D detected the highest proportions (79% and 71%), whereas Guineu and jAligner4GCxGC performed identically (68% and 33%). Alignment-related processing required seconds for Guineu, 4 min for jAligner4GCxGC, and 9 min for Sync 2D. However, Sync 2D provided the quickest end-to-end workflow. These results highlight practical trade-offs between commercial and open-source workflows and demonstrate that jAligner4GCxGC provides a transparent and reproducible solution for GC × GC–MS peak alignment.

## Introduction

Comprehensive two-dimensional gas chromatography–mass spectrometry (GC × GC–MS) has emerged as a powerful analytical technique for detecting individual compounds in multiresidue chemical mixtures derived from complex matrices such as body fluids [1], dust [2], petroleum [3], or edible oils [4], enabling both target analysis and non-target screening (NTS) in diverse applications including metabolomics, environmental monitoring, petrochemical analysis, and food science [5, 6]. Its exceptional chromatographic resolution and sensitivity surpass those of conventional one-dimensional approaches. However, GC × GC–MS datasets are highly complex and thus difficult to interpret, which hinders the broader method’s adoption. These difficulties are especially pronounced when performing peak alignment based on the inter-sample retention time (RT) [7, 8].

In terms of alignment, the ultimate goal of many GC × GC–MS studies (including this one) is to generate a so-called alignment result table (ART) in which all unique individual chemical features/compounds detected across a sample set are linked to the corresponding peak area/concentration data for all of the studied samples, making it possible to easily obtain an overview of the results, perform inter-sample comparisons, and establish frequency-based prioritizations for subsequent data analyses. GC × GC–MS experiments generate large, multidimensional datasets that impose heavy computational demands. Critical steps in data processing include peak detection, deconvolution, compound identification, and especially RT-based peak alignment, which ensures that the same chemical feature is matched/compared across multiple chromatograms. Without accurate alignment, minor retention time shifts can cause mismatching of peaks leading to incorrect compound identification and quantification, which in turn compromises comparability and reproducibility [9–11].

Several computational strategies have been proposed to address these challenges, using both spectral-dependent and spectral-independent algorithms including DIstance and Spectrum Correlation Optimization (DISCO) [12], Bidirectional best-hit Peak Assignment and Clique Extension for 2D chromatograms (BiPACE 2D) [13], MSort [14], 2-Dimensional Correlation Optimized Warping (2-D COW) [15], piecewise alignment methods rooted in chemometric strategies [16], and tile-based comparison techniques [17]. However, many implementations are limited to proprietary commercial software.

A comprehensive listing of existing GC × GC–MS data processing solutions, including commercial, bespoke, and open-source software, was provided by Wilde *et al*. (2020) [18]. Although this compilation is not exhaustive, it provides critical contextual information on the evolution of alignment methodologies and highlights the ongoing need for expanded evaluation of available tools.

Building on this foundation, the recent development of GcDUO [19], an open-source software package implemented in R and designed for modular processing, represents a significant methodological advance in GC × GC–MS data analysis [19]. GcDUO integrates tensor-decomposition chemometric techniques, namely PARAllel FACtor analysis (PARAFAC) [20] and its more flexible variant PARAFAC2 [21], into a modular workflow that includes raw data import, region-of-interest (ROI) selection, deconvolution, peak annotation, batch data integration, and visualization. PARAFAC effectively deconvolutes overlapping peaks by assuming trilinearity in chromatographic-spectral data, while PARAFAC2 relaxes this constraint to accommodate RT shifts across runs [19]. However, GcDUO does not generate ART, hence, it was not included in this study.

Reflecting the ongoing expansion of the GC × GC–MS alignment software ecosystem, eleven representative commercial programs and open-source applications, scripts, and algorithms were considered for inclusion in this study: DA_2DCHROM [22], GC Automation [18], GC2MS [23], Guineu [1], MetPP [24], MOREPEAKS [25], R2DGC [26], RGC×GC [27], RMet [28], ChromaTOF Sync 2D (LECO Corp.) [29], and GasPedal (DECODON) [30]. Tile-based applications such as ChromaTOF Tile (LECO Corp.) [31], were excluded because they analyse chromatographic data by dividing it into predefined RT regions (tiles) and perform statistical comparisons within tiles instead of tracking individual compounds or peaks. This means they cannot generate an ART, which was a core requirement for evaluating alignment performance in this work.

While 11 tools were initially considered for inclusion, 9 were ultimately excluded for reasons including (i) inability to read raw or converted input files generated during the study, (ii) only being able perform alignments for a limited number of samples, e.g. two, and (iii) having a demo license with limited functionality; see Table S1 in the Supplementary Information (SI), available at https://doi.org/10.5281/zenodo.20510279 for more details. In-depth benchmarking was thus performed with two applications offering distinct capabilities: Guineu and ChromaTOF Sync 2D. Guineu has been selected as it is an open-source tool (developed in Java for metabolomics GC × GC-TOFMS data [1]), while ChromaTOF Sync 2D is vendor-specific software supplied by the dominant GC × GC–MS hardware manufacturer (LECO Corp.) that offers synchronized alignment and deconvolution across grouped GC × GC–MS sample sets [29]. Both tools generate ARTs. These applications were evaluated alongside jAligner4GCxGC, a custom alignment solution developed in-house using the Julia programming language. The three applications were compared in terms of speed, alignment accuracy, ease of use, and transparency.

Julia combines dynamic language flexibility, fast just-in-time compilation, efficient numerical operations, and extensive scientific libraries, making it a suitable platform for developing high-throughput, reproducible mass spectrometry data processing pipelines [32]. Notably, the Julia ecosystem includes toolsets such as jHRMSToolBox that offers modular workflows for feature detection, alignment, visualization, and statistical evaluation across diverse HRMS platforms [33]. jAligner4GCxGC was developed to deliver a fast, accurate, and transparent alignment framework that is explicitly tailored to GC × GC–MS data.

This study aims to highlight the strengths, limitations, and optimal use areas of applications across the GC × GC–MS alignment landscape, including both commercial and open-source solutions as well as a new solution for inter-sample GC × GC–MS peak alignment, data curation, and NTS metadata collection. Such performance assessments will play a key role in driving wider acceptance and use of GC × GC–MS.

## Materials and methods

### Chemicals and reagents

SupraSolv grade (for gas chromatography) acetone, dichloromethane (DCM), *n*-hexane, methanol, and toluene were purchased from Merck KGaA (Darmstadt, Germany). For alignment performance evaluation, 8270 MegaMix (containing 76 volatile and semi-volatile organic compounds (VOCs/SVOCs)), SVOC Additions Standard (containing 23 SVOCs), and Terpenes MegaMix #1 (containing 43 terpene-related compounds) were purchased from Restek Corp. (Bellefonte, PA, USA) and combined into a single reference standard mixture along with two in-house mixtures of personal care product chemicals (8 compounds) and phenolic compounds (9 compounds); see Table S2 in the SI (available at https://doi.org/10.5281/zenodo.20510279). An alkane mixture containing C7–C40 *n*-alkanes was purchased from Supelco Inc. (Bellefonte, PA, USA) and used for linear retention index (RI) calculation (SI, Table S3).

### Samples

The software evaluation (SI, Table S4) used three dust samples: vacuum-cleaner dust (sieved to <125 μm) from an apartment in Umeå, Sweden (UMPS); a composite sample from the NORMAN Network European dust trial [2] (EUCS); and a North American Standard Reference Material sample ‘SRM 2585 – Organic Contaminants in House Dust’ from the National Institute of Standards and Technology (NIST). A procedural blank (BLNK) was also included.

### Extraction, clean-up, and fractionation

Before use, all glassware was cleaned in a dishwasher and baked overnight in a muffle furnace at 550 °C. Approximately 50 mg of each dust sample was weighed in 14-ml screw-cap glass bottles and extracted using the protocol of Moschet *et al*. (2018) [34] with slight modifications. First, 3 ml *n*-hexane:acetone (3:1, v/v) was added to the samples and they were vortexed for 1 min before being ultrasonicated for 15 min and then centrifuged at 3500 rpm for 5 min. The supernatants were collected in Büchi graduated glass vials (residual volume: 0.3 ml). Next, 3 ml of acetone was added to the remaining dust and the procedure was repeated. The supernatants were collected and combined with the previous ones and the combined extracts were evaporated using a Büchi SyncorePlus Analyst (Büchi Labortechnik GmbH, Essen, Germany) until ~0.3 ml remained. Then, the extracts were passed through an ISOLUTE NH2 solid-phase extraction (SPE) cartridge (500 mg, 6 mL; Biotage, Uppsala, Sweden) to remove fatty acids and other polar compounds. It should be noted that the NH2 SPE cleanup also removes some spiked compounds such as dinitrophenols. The SPE cartridges were preconditioned with 5 ml of dichloromethane (DCM) using the Büchi SyncorePlus Analyst’s SPE setup and a gentle vacuum. Next, the extracts were loaded onto the SPE cartridges and eluted with 20 ml of DCM, again applying a gentle vacuum. Finally, the extracts were evaporated and the solvent was exchanged to toluene using Büchi SyncorePlus Analyst. The purified samples were transferred to GC vials and their volumes were adjusted to 1 ml. The procedural blank was run in parallel to the dust samples.

For alignment software performance evaluation, 20-μl aliquots of the three samples and the blank were taken and spiked with 20 μl of the reference standard mixture at three concentration levels: ca. 0.5, 5, and 50 pg per 1-μl injection (SI, Table S2).

### Two-dimensional gas chromatography–time-of-flight mass spectrometry (GC × GC-TOFMS)

Analyses were performed with an Agilent 8890 GC System (Agilent Technologies, Santa Clara, CA, USA) coupled to a LECO Pegasus BT 4D GC × GC-TOFMS (LECO Corp., St. Joseph, MI, USA). A secondary oven and a consumable-free modulator quad-jet dual stage thermal modulator (trapping at −80 °C) were built into the main GC oven. Two serially connected 30 m × 0.25 mm × 0.25 μm 5%-phenyl methylpolysiloxane columns (one HP-5 ms and one DB-5 ms-UI; Agilent Technologies, Santa Clara, CA, USA) were used for the first-dimension separation and a 2 m × 0.25 mm × 0.25 μm 35%-trifluoropropyl methylpolysiloxane column (Rtx-200; Restek, Bellefonte, PA, USA) was used for the second-dimension separation (see Method S1 in the SI available online at http://bib.oxfordjournals.org/ for details). Helium was used as the carrier gas at a constant flow rate of 1.6 mL/min.

Samples were injected splitlessly (1 μl) at 280 °C. The primary GC oven was initially maintained at 80 °C for 1 min, then ramped at 3 °C/min to 320 °C, which was held for 5 min, before ramping to 240 °C at 40 °C/min and holding for another 5 min. The secondary oven had an offset of +5 °C relative to the first oven and the modulator had an offset of +15 °C relative to the secondary oven. The transfer line temperature was 320 °C. The modulation timing was set as follows: (i) start of run to 1300 s: 2.50 s (second dimension time), 0.40 s (hot pulse time), 0.85 s (cool time between stages), followed by (ii) 1300 s to end of run: 2.50 s (second dimension time), 0.75 s (hot pulse time), 0.50 s (cool time between stages). See Method S1 in the SI available online at http://bib.oxfordjournals.org/ for details. Source and analyser focus, mass calibration, gain optimization, and tune validation were run at the beginning of the sample batch. Then, two injections of toluene were performed before the sample injections.

Analyses were performed in electron ionization (EI) mode at 70 eV electron energy, 1.0 μA emission current, and an ion source temperature of 250 °C. Data were stored over the mass range of 47–750 amu, at an acquisition rate of 100 spectra/s. The system was tuned using perfluorotributylamine.

Procedural blanks, reference standard mixture, and other quality control samples were acquired in parallel to the dust samples. Additionally, a mixture of C7–C40 *n*-alkanes in toluene was injected to enable RI calculations. Data were acquired using ChromaTOF Software for Pegasus BT (v5.51.50.0; LECO Corp., St. Joseph, MI, USA).

### Data processing for alignment software performance evaluation

**Figure 1:**
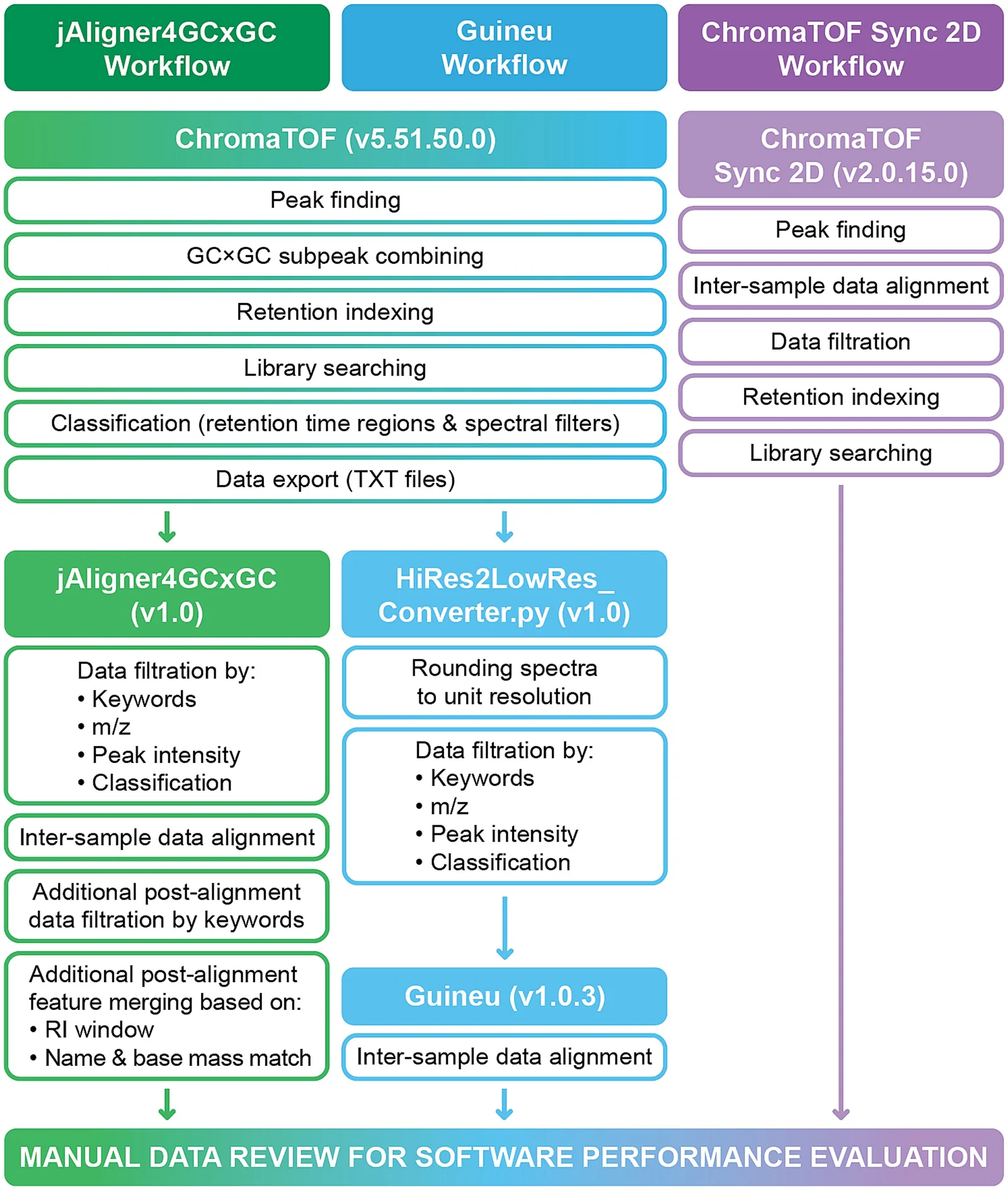
Overview of data processing workflows for three inter-sample peak alignment solutions: jAligner4GCxGC, Guineu, and ChromaTOF Sync 2D.

Over 20 GC × GC–MS applications were initially screened for inter-sample peak alignment, of which 11 were found to be compatible with the data sets generated during this work (CSV/TXT, LECO Sample File (SMP), mzML, or netCDF), see Table S1 in the SI. Three solutions were selected for workflow development and in-depth evaluation: Guineu (v1.0.3; Espoo, Finland [1]), ChromaTOF Sync 2D (v2.0.15.0; Leco Corp., St Joseph, MI, USA), and jAligner4GCxGC, which was developed by the authors (available at https://github.com/r3bryk/jAligner4GCxGC). The main steps in these data processing workflows were peak detection, inter-sample alignment, and detection of spiked compounds. Figure 1 shows generalized outlines of the workflows developed and used in this study; the workflows and associated parameters are described comprehensively in the SI (Workflows S1-S3 available online at http://bib.oxfordjournals.org/) and the specifications of the device used in this study are provided in Table S7. The primary objective of this benchmarking was to compare complete analytical workflows as they would typically be used by end users. Because the evaluated software packages differ in their peak detection, deconvolution, and preprocessing strategies, particularly Sync 2D *versus* the ChromaTOF-based open-source workflows, the comparison cannot completely isolate differences attributable solely to the alignment algorithms. Consequently, comparisons involving Sync 2D should be interpreted as evaluations of practical workflow performance rather than controlled algorithm-to-algorithm benchmarks. In contrast, comparisons between Guineu and jAligner4GCxGC are less affected by upstream processing because both workflows use identical ChromaTOF-generated inputs.

As far as possible, comparable processing conditions were used across workflows. Nevertheless, complete harmonization was not feasible as the software packages differ in required input formats, preprocessing strategies, and available parameterization. Consequently, identical alignment conditions could not always be reproduced across all platforms. When identical settings could not be maintained across software packages, settings were either selected based on prior experience with each application or optimized empirically to achieve optimal alignment. The settings chosen by the authors do not necessarily reflect default/recommended settings for each software package. See Workflows S1-S3 in the SI available online at http://bib.oxfordjournals.org/ for more information about the settings. It should be noted that most of the evaluations other than those of Sync 2D were performed during the summer of 2023, so the software packages involved may have received updates or changes, or been discontinued, in the three years since then. Even though Sync 2D was evaluated in 2025, the version used (v2.0.15.0) was already outdated by the time of writing.

The jAligner4GCxGC and Guineu workflows use ChromaTOF for peak picking, deconvolution, peak area determination, retention indexing, and spectral library searching (using the NIST 2023 EI mass spectral library; National Institute of Standards and Technology, Gaithersburg, MD, USA). Sync 2D has built-in functionalities for these tasks, which is one of the main ways it differs from the two open-source applications. In addition, compounds belonging to the chemical classes, such as alkane, column bleed, toluene, DCM, phthalate, or DINCH (1,2-cyclohexane dicarboxylic acid diisononyl esters), were sorted into the respective class using chromatographic regions and spectral peak filters in ChromaTOF. Next, the resulting sample data were exported as tab-delimited TXT files.

In the jAligner4GCxGC workflow, the exported data were preprocessed, aligned, and postprocessed using the application. Preprocessing involved four filtration steps: (i) removal of compounds with names containing specific keywords, e.g. ‘siloxane’, (ii) removal of compounds whose spectra contain *m/z* peaks corresponding to that of toluene (*m/z* 91 or 92) with no higher *m/z* values, (iii) removal of low intensity (<3%) ions from the spectra, and (iv) removal of compounds classified as bleed, toluene, or DCM in ChromaTOF. Then, the samples were aligned using the classic DISCO algorithm [12], and additional post-alignment keyword-based filtration was performed. Next, an additional feature merging step was performed to avoid splitting of broad peaks. This step merged replicate features having identical names and base masses (for their most intense fragments) into a single feature if they eluted close to one-another (e.g. if their ΔRI was <7). Finally, the alignment result table (ART) was exported as a CSV file (SI, Table S9, available at https://doi.org/10.5281/zenodo.20510279).

For the Guineu workflow, which can handle only unit-resolution data, data preprocessing was performed using the HiRes2LowRes_Converter.py script (available at https://github.com/r3bryk/HiRes2LowRes_Converter), which was developed in-house using Python v3.11 (Python Software Foundation, Wilmington, DE, USA). This script converts GC × GC spectra into unit resolution format and then performs the four data filtration steps described in the jAligner4GCxGC workflow. Subsequent inter-sample GC × GC peak alignment was performed with Guineu. Finally, the ART was exported as a CSV file.

For the ChromaTOF Sync 2D workflow, every processing step was performed in the application itself, including: (i) peak finding, (ii) inter-sample peak alignment, (iii) data filtration, (iv) retention indexing, and (v) library searching using the NIST 2023 EI mass spectral library. The ART was then exported as a tab-separated values (TSV) file. The only step performed outside the application was the calculation of detection frequencies. ChromaTOF Sync 2D almost never returns a peak area value of 0 in the ART, which can confound detection frequency estimation. To address this issue, a histogram plot for the distribution of log10-transformed peak areas was created and evaluated. A bimodal distribution was observed, with an intersection between the two clusters at ca. 10^4^ (SI, Workflow S3 available online at http://bib.oxfordjournals.org/), which is equivalent to 10,000 peak area units. On this basis, all area values below 10,000 were replaced with 0.

### Full data processing workflows

**Figure 2:**
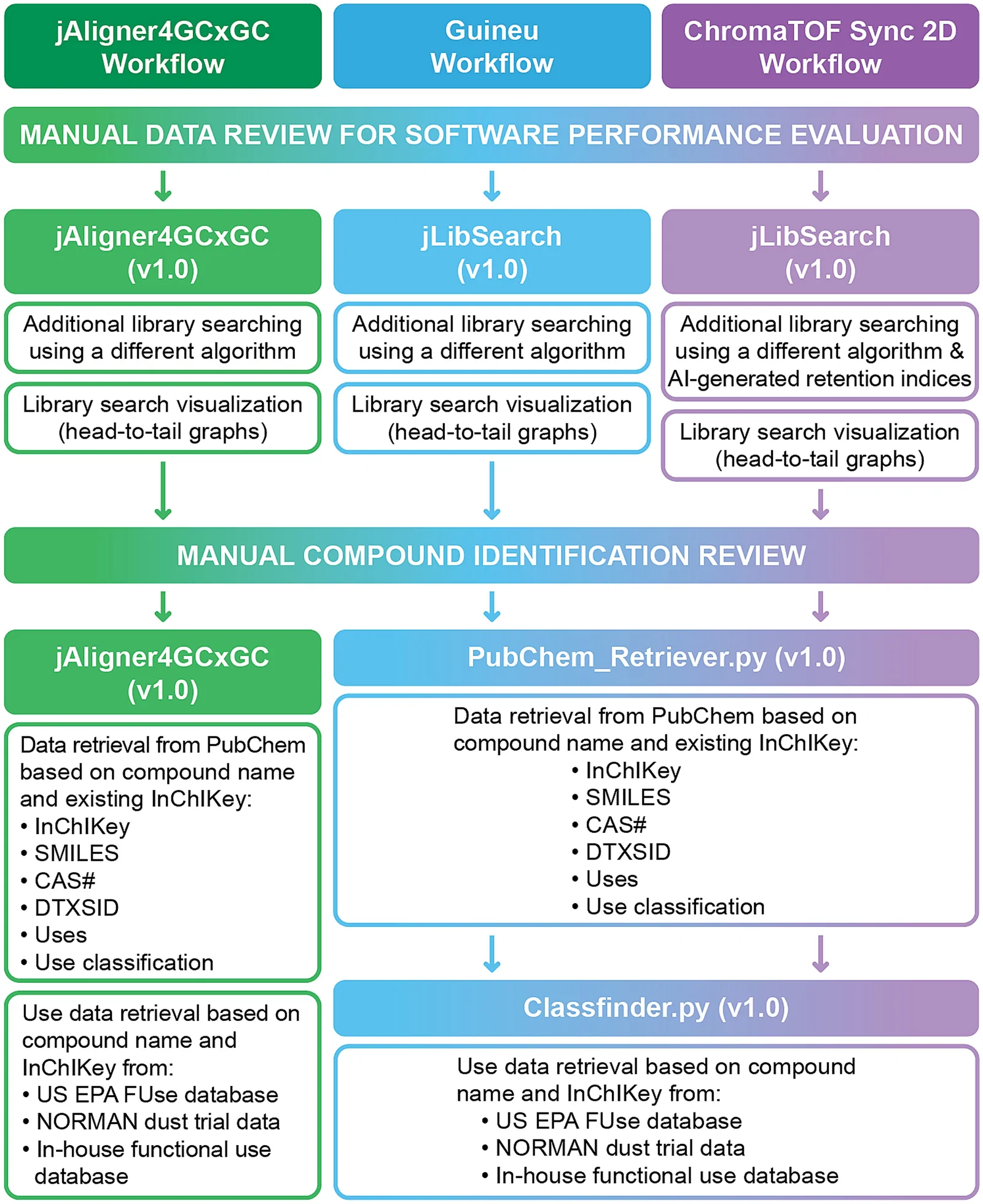
Proposed information retrieval steps and tools for full non-target screening data processing using the newly developed jAligner4GCxGC, Guineu, and ChromaTOF Sync 2D workflows.

While this study’s main aim was to evaluate different peak alignment software packages and develop a new open-source GC × GC–MS peak alignment solution, full NTS-based data processing workflows were developed for each of the three solutions that were evaluated (Fig. 2).

Proposed further steps include (i) library searching for consensus spectra using a different algorithm (DISCO or normalized dot product (NDP)) or additional in-house or commercial EI spectral databases, (ii) library search visualization using head-to-tail graphs for data review, (iii) retrieval of compound identifiers such as InChIKey, SMILES, CAS#, and DTXSID as well as Uses and Use Classification data from PubChem [35], and (iv) compound classification using data from, e.g. the EPA Functional Use (FUse) Database [36], NORMAN European dust trial data [37], or an available in-house database. All steps in the jAligner4GCxGC workflow were performed using built-in functions. New Julia and Python 3 scripts were developed for the other workflows, including jLibSearch (available at https://github.com/r3bryk/jLibSearch), PubChem_Retriever.py (available at https://github.com/r3bryk/PubChem_Retriever), and Classfinder.py (available at https://github.com/r3bryk/Classfinder).

## Results and discussion

### Performance evaluations

#### General conditions for performance evaluation

After processing using the three software workflows, 10 496 features were initially detected with jAligner4GCxGC, 11 195 with Guineu, and 8406 with Sync 2D. The ARTs were examined manually to determine the number of spiked compounds that were both detected and correctly aligned across samples. A compound was considered correctly detected if the ART included a peak with the correct library identification. The identification was accepted if the correct compound was the top library hit or, for features with high spectral similarity, if an isomeric compound was detected and subsequently reassigned the correct identity *via* manual review. The identities were verified based on RI and spectral matches. A compound was considered aligned if the same feature was matched across all samples based on predefined RT/RI windows and spectral similarity thresholds. Minor deviations within these tolerances (e.g. slightly wider RT/RI windows) were not considered misalignments. Detected compounds were only included in the performance evaluation if they had quantifiable peak areas above the defined detection threshold (meaning an area > 10 000 for Sync 2D). The overall performance of each application was then quantified as the percentage of spiked compounds present in the ART relative to the number of spiked compounds falling within the GC method’s experimental domain (151 in total; SI, Table S2).

The number of compounds aligned automatically using each workflow was checked, then replicates were manually merged into one feature when necessary. Since the numbers of compounds detected automatically by each application did not differ appreciably from those obtained after manual peak merging, only the latter are discussed.

**Figure 3:**
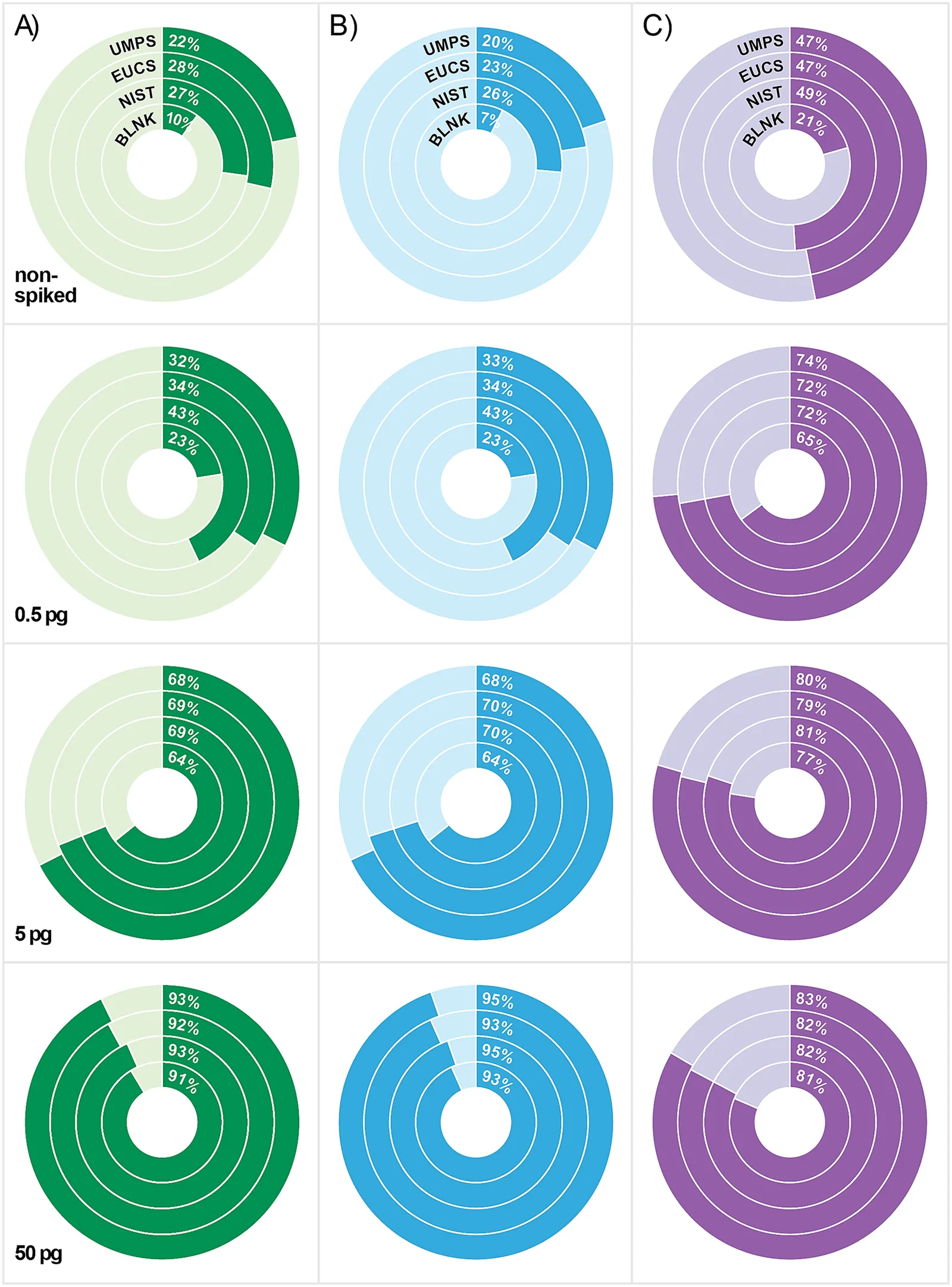
Percentages of reference compounds detected after alignment of peak lists for UMPS, EUCS, NIST, and BLNK samples. The top row shows the numbers of reference compounds detected in non-spiked dust samples using the jAligner4GCxGC (column A), Guineu (column B), and ChromaTOF Sync 2D (column C) workflows. The remaining rows show the corresponding results for samples spiked at concentrations of 0.5, 5, and 50 pg/μl.

No attempt was made to correct for the background abundance of compounds naturally present in the indoor dust matrix because determination of actual concentrations was not a goal of this study and their presence would not influence peak alignment results. These analytes were included in the evaluation without adjustment, using the same detection and alignment criteria as all other spiked compounds. However, to illustrate the natural presence of the reference compounds in household dust, Fig. 3 (row ‘non-spiked’) shows the number of reference compounds detected in blank (BLNK) and non-spiked dust samples after alignment for each application. These numbers are expressed as percentages of the total number of reference compounds. For blank samples, the proportion of detected compounds ranged from 7% with Guineu to 21% with Sync 2D. As expected, considerably more reference compounds were detected in non-spiked dust samples. jAligner4GCxGC and Guineu performed similarly for these samples, detecting roughly one-fourth (20% to 28%) of the reference compounds in the NORMAN dust trial composite dust [2] (EUCS), NIST SRM dust (NIST), and Umeå house dust samples (UMPS). Sync 2D detected twice as many reference compounds (47% to 49%), possibly because it is better at detecting and correctly assigning low-abundance analytes.

#### Number of detected spiked compounds

As shown in Fig. 3, the number of compounds detected after aligning samples spiked at 0.5 pg/μl was highest for ChromaTOF Sync 2D (65%–74%, 71% on average), while jAligner4GCxGC and Guineu both detected 23%–43% of the reference compounds (33% on average), likely because the same input peak list was used in both cases.

For 5 pg/μl samples, Sync 2D again offered the best performance (77%–81%, 79% on average) while jAligner4GCxGC and Guineu performed similarly, with detection percentages of 64%–70% (68% on average) and 64%–69% (68% on average), respectively.

A different outcome was obtained for samples spiked at 50 pg/μl: Sync 2D had the lowest detection percentages, at 81%–83% (82% on average), while the other two applications found over 90% of the spiked compounds: the detection percentages for jAligner4GCxGC and Guineu were 91%–93% (92% on average) and 93%–95% (94% on average), respectively.

In general, Guineu found 96% of the spiked compound list across all samples, jAligner4GCxGC found 95%, and Sync 2D found 86%. None of the applications successfully detected compounds such as *α*-pinene, camphene, (−)-*β*-pinene, 3,3′-dichlorobenzidine, 2,4-dinitrophenol, or 4,6-dinitro-2-methylphenol. The first two terpenes elute early in the chromatographic run, close to the acquisition onset, and may therefore be partially or entirely missed in most samples. The third terpene, (−)-*β*-pinene, co-eluted with sabinene, may have caused misalignment, as discussed in Section *Overall impressions of the evaluated software packages*. The last three compounds are difficult to analyse by GC–MS as they are acidic and have low response factors.

Sync 2D has high sensitivity to weak signals and thus detected several compounds missed by the other applications, including the dinitrophenolic pesticide dinoseb. This is notable because the concentration of dinitrophenol derivatives in the extracts would have been greatly reduced during the SPE cleanup stage.

In all workflows, misalignments could occasionally arise from RT/RI shifts outside the defined tolerance windows or high spectral similarity between closely eluting compounds. These effects were most pronounced for chromatographic regions containing many co-eluting compounds with similar spectra that are difficult to cleanup, notably early eluting volatiles such as terpenes as well as high-molecular-weight phthalates at the end of chromatographic run.

#### Time needed for processing

The alignment speed for the 12 samples, reference mixture, and QC samples varied substantially across the evaluated workflows. Guineu required only seconds to complete alignment. However, an additional step is required to convert files into a Guineu-compatible format using HiRes2LowRes_Converter.py, a step not needed for the other tools. For jAligner4GCxGC, data preprocessing-independent alignment operations (including pre-alignment filtration, alignment, and post-alignment filtration and feature merging) required 4 min 21 s (SI, Table S5A). For ChromaTOF Sync 2D, the corresponding alignment and post-alignment steps required 8 min 50 s (SI, Table S6). Although these measurements still reflect differences in workflow implementation, excluding upstream preprocessing reduces one important source of variation and provides a closer approximation of the computational cost associated with alignment-related operations.

Importantly, in the Guineu and jAligner4GCxGC workflows, post-acquisition data preprocessing is performed externally in ChromaTOF (SI, Table S5B). While this step can be automated and run unattended, it remains computationally expensive, primarily due to library search being performed for each individual sample (ca. 21 min per sample, ca. 6 h per batch). In contrast, Sync 2D performs library searching only once on the final ART, substantially reducing total processing time (7 min 46 s for the batch; SI, Table S6).

Separating preprocessing from alignment-related operations improves the fairness of computational runtime comparisons, although complete separation is not possible because Sync 2D uses a fundamentally different integrated processing pipeline. When alignment-related operations are considered separately, jAligner4GCxGC required less computational time than Sync 2D under the evaluated conditions. However, when end-to-end analytical workflows are considered, Sync 2D completed the overall processing more rapidly owing to its integrated preprocessing and single library searching step. For small sample batches like that used here, all three solutions provide acceptable turnaround times, while for larger sample sets, the greater speed of Sync 2D would be useful.

### Advantages and drawbacks of jAligner4GCxGC

Unlike the other two applications, jAligner4GCxGC currently lacks a graphic user interface (GUI). However, it can be easily executed in a Visual Studio Code notebook (Microsoft Corp., Redmond, WA, USA) that will display messages for each step, including any error messages that arise.

**Figure 4:**
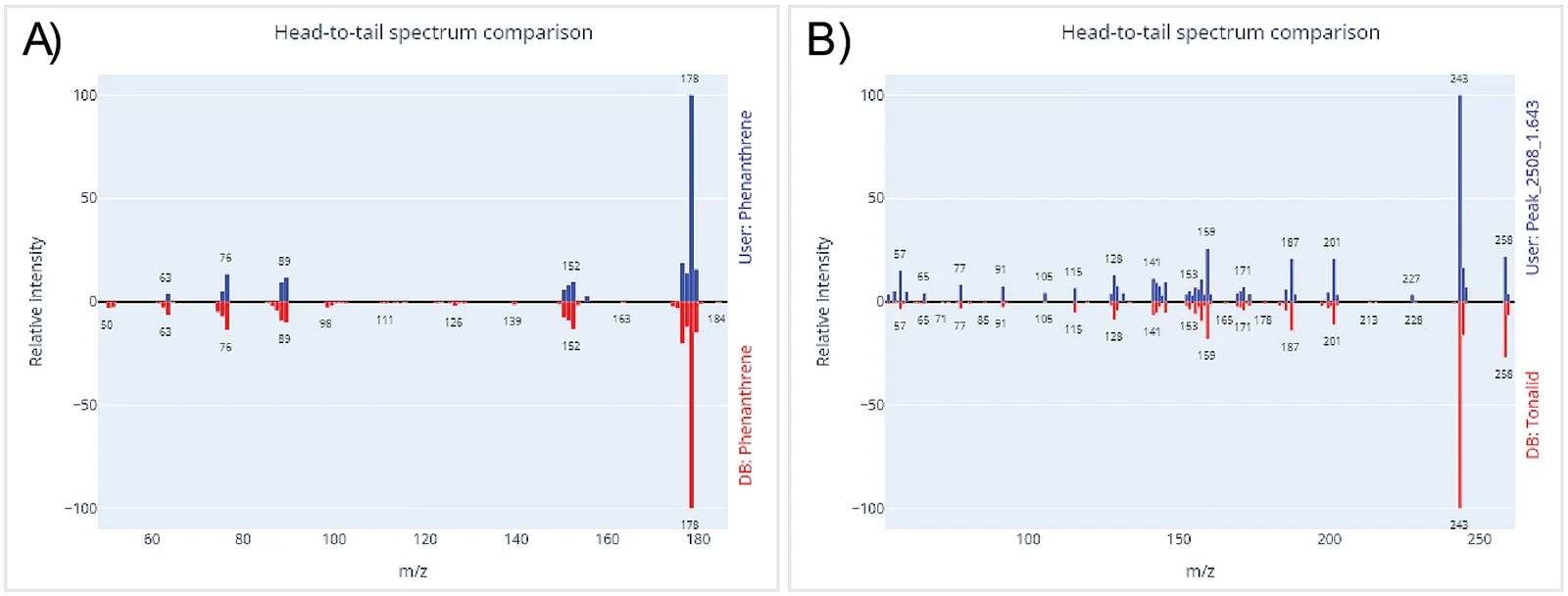
Head-to-tail (user-to-database) spectrum comparison graphs showing results obtained with the library search functionality of jAligner4GCxGC. A) Additional structure confirmation for phenanthrene. B) Identification of an unknown feature as tonalid.

In addition, jAligner4GCxGC includes built-in functionalities for performing additional library searches, compound data retrieval, and compound classification (for a full description, see https://github.com/r3bryk/jAligner4GCxGC). The retrieved data can help, e.g., aggregate identified compounds into functional use classes and identify dominating country-specific compound classes. The additional library search functionality uses alternative spectral matching algorithms (DISCO or NDP) and may be of particular interest because it could yield different library search results to the algorithms used by other software. jAligner4GCxGC uses the EI spectrum of the richest sample in the sample batch, which can sometimes lead to better library matches and enable identification of previously unassigned features (see Fig. 4).

Moreover, the integrated data retrieval functionality can be used to gather useful metadata including chemical identifiers and functional use data from online and in-house databases. Another – and maybe the main – advantage of jAligner4GCxGC is that it is a free, open-source package, meaning that users with programming skills can modify any part of it and add new functionalities, including machine learning (ML)-based approaches.

A major disadvantage of open-source solutions like jAligner4GCxGC is that they must be maintained and updated regularly because programming languages and associated packages evolve over time, potentially leading to compatibility issues or code obsolescence. To mitigate this, jAligner4GCxGC has been implemented as a Julia package with fully specified Project.toml and Manifest.toml files, which define the exact versions of all script dependencies during installation. This ensures a stable computational environment and long-term usability, eliminating issues arising from package updates or deprecations.

### Full data processing workflows and additional functionalities

All the above-mentioned jAligner4GCxGC functionalities were also implemented in several separate Julia and Python scripts (see section 2.6) to complement the two other software packages and create full NTS data processing workflows. In the Guineu workflow, which also uses ChromaTOF in its first step, alignment is performed with Guineu while custom scripts are used for data preprocessing (i.e., converting mass spectra to unit resolution) as well as additional library searching and post-alignment data retrieval (Figs 1 and 2). Sync 2D ART files can be complemented with post-alignment data processing to eliminate false positives (see section 2.5). In addition, the library search functionality in the evaluated version of Sync 2D does not permit the use of estimated semi-standard non-polar or AI-predicted semi-standard non-polar RI values from the NIST 2023 library, which occasionally results in incorrect compound identifications. The full Sync 2D workflow therefore requires additional library searching and post-alignment data retrieval procedures using custom scripts (Fig. 2).

### Overall impressions of the evaluated software packages

Each of the evaluated solutions has advantages and disadvantages. For instance, ChromaTOF Sync 2D can generate various plots that are important for data overview and review, such as compound spectra and 2D and 3D chromatograms. Unfortunately, jAligner4GCxGC and Guineu lack comparable functionality.

The main advantage of Guineu is that it performs alignment very quickly, usually taking no more than a few minutes at most. Moreover, it is free software. However, Guineu has difficulties aligning large datasets with several hundred samples or more, leading to ARTs that contain many replicate features.

A major advantage of Sync 2D is that it detects features even when the corresponding analyte is present at very low concentrations (see Fig. 3, row 0.5 pg). For samples spiked at 0.5 pg/μL, Sync 2D picked up >50% more compounds than the second-best software package. However, its performance did not improve drastically at higher spiking levels under the conditions applied in this work (see Fig. 3, row 50 pg). Sync 2D also has valuable built-in functionalities for statistical analysis using PCA as well as various simple and advanced filters. Its disadvantages include a relatively high false positive rate (section 2.5) and the presence of duplicate ART features, likely because of weaknesses in the current implementation of the alignment algorithm. This may change in the future, after the implementation of user feedback; it should be noted that the version of the software evaluated in this work (v2.0.15.0) had become outdated by the time of writing. Duplicate features in the ART can be merged manually using the Merge functionality in Sync 2D ART.

Like Guineu and Sync 2D, jAligner4GCxGC returns replicate features that can be merged automatically using a built-in function based on exact name and base mass matches within a specified RI tolerance window. However, sometimes spectra representing the same compound in different samples have different base masses, either as a consequence of compromised deconvolution (for example, the base mass may be an alkane peak embedded in the spectrum, e.g., around *m/z* 57) or because the most intense peak in a cluster differs between samples (e.g., *m/z* 146 *versus* 148 for dichlorobenzenes). This can cause replicates to be retained after post-alignment merging. Misalignment may also occur if the preceding library search resulted in a misidentified compound, i.e., the wrong name was assigned to a compound similar or isomeric to the one in question, as discussed in section 3.5 (see also Table S8, Fig. S1, and Fig. S2 in the SI).

### General issues with peak alignment

The main unsolved problems in inter-sample GC × GC–MS peak alignment for complex matrices such as indoor dust appear to concern the handling of closely eluting compounds and retention shifts between samples or batches. This is especially true for compounds that have similar spectra (including the same base mass) and elute close to each other, such as terpenes. If the specified RT/RI recognition window is too wide, these features/compounds will be merged into one feature/compound. Conversely, if the RT/RI window is too narrow, the ART will contain multiple entries representing the same feature/compound (i.e., replicates). Cross-feature alignment is also possible, meaning that feature A gets mixed up with feature B in different samples, resulting in data gaps or inflated peak areas in the ART.

For example, the NIST 2023 EI mass spectral library includes 162 compounds with a molecular weight of 136 and the formula C_10_H_16_. Nine of these were present in the spiked reference standard mixture. Of these, *α*-pinene and camphene elute within 15 RI units (semi-standard non-polar RI) of one-another; the ΔRI for sabinene and (−)-*β*-pinene is 4 RI units, while that for *α*-phellandrene and 3-carene is 6 RI units, and 18 RI units separate γ-terpinene and terpinolene (SI, Table S8). All these compounds have a base mass of 93 and quite similar spectra. In addition, sabinene and (−)-*β*-pinene co-elute (at least in the 1^st^ dimension) with aniline, another spiked compound that has a base mass of 93 (see Fig. S1 in the SI) and may be aligned with any of these two compounds or other terpenes in the samples if a low spectral similarity threshold or a wide RT2 tolerance window are chosen (SI, Fig. S2). Accordingly, none of the alignment tools returned all three of these compounds: only aniline and one of the terpenes were present in every ART under the tested conditions. This outcome highlights a general limitation of automated peak alignment in complex mixtures: when multiple compounds share the same base mass, have quite similar spectra, and elute in close proximity, even small changes in spectral similarity thresholds or RT/RI tolerances can cause misassignments. To make matters worse, peaks tend to broaden in both chromatographic dimensions over time (SI, Fig. S1).

To address this problem, sample data files can be split into distinct chromatographic regions (easily done for netCDF files in ChromaTOF and for TXT files either in Excel or using a custom script) and aligned separately using various retention windows and spectral similarity thresholds. This enables the use of narrower windows and higher spectral similarity thresholds for terpenes and other early eluting compounds together with wider windows towards the end of the chromatographic run.

## Conclusions

This study introduces jAligner4GCxGC, a new open-access and open-source Julia package for inter-sample GC × GC–MS peak alignment. Its performance has been evaluated against two alternative software packages for this purpose. The results obtained show that free open-source software packages can provide performance comparable to established commercial workflows for the evaluated datasets while offering substantially greater methodological transparency. In the evaluated workflow benchmarks, the two open-source applications (Guineu and jAligner4GCxGC) recovered similar or higher proportions of correctly aligned spiked compounds than the commercial software ChromaTOF Sync 2D when capturing reference compounds spiked at concentrations of 5 pg/μL and 50 pg/μL, whereas Sync 2D performed better at 0.5 pg/μL. Because the evaluated workflows differ in upstream peak detection and preprocessing, these observations should be interpreted as workflow-level comparisons rather than evidence of superior alignment algorithms alone.

Additional built-in functionalities and the potential to modify the application to suit specific needs make jAligner4GCxGC a good solution for GC × GC–MS based NTS of environmental samples. However, the commercial software package Sync 2D may offer valuable advantages when processing large numbers of similar samples with relatively high analyte concentrations (as may be necessary in metabolomics projects among other things) because it includes integrated statistical tools, such as PCA, for exploring inter-sample and inter-feature relationships. It is worth noting, however, that Sync 2D can only be used by owners of LECO instruments.

The main challenge in inter-sample GC × GC–MS peak alignment for complex environmental matrices probably lies in misalignment of features across samples, especially for compounds with similar spectra such as terpenes. Tweaking alignment parameters such as RT windows and spectra similarity thresholds may yield more reliable alignments. It can also be helpful to split the initial chromatograms into multiple regions and apply different parameters to each region.

The limitation of this study is that identical upstream preprocessing could not be applied across all evaluated software packages because Sync 2D uses proprietary integrated peak detection and deconvolution procedures. Although preprocessing-independent runtimes were reported where possible, complete isolation of alignment performance was not feasible.

Another important challenge is that there is little public information about the algorithms used in commercial software and their source code is generally inaccessible. Without understanding what is happening, it can be difficult to optimize peak picking parameters to improve the software’s performance. This challenge may be overcome through cooperation between software developers and users, which can be time-consuming and sometimes complicated but is always worth trying.

**Key Points**

- GC × GC–MS adoption limited by lack of transparent, robust inter-sample peak alignment workflows for processing of its complex data.
- jAligner4GCxGC is an open-source package developed in Julia that provides GC × GC–MS alignment with integrated dataset preprocessing, postprocessing, and library searching.
- Performance benchmarked against Guineu (open-source) and ChromaTOF Sync 2D (commercial) software packages.
- Spike-recovery evaluation shows competitive performance across concentration levels and variable alignment speed trade-offs.
- jAligner4GCxGC delivers a fully transparent, reproducible, and effective alignment workflow.

## Supplementary Material

Supplementary_material_bbag458

## Data Availability

The data underlying this article are available in Zenodo, at https://doi.org/10.5281/zenodo.18833682.
