# Supplementary material for: Evaluation of peak alignment performance of commercial and open-source tools for GC×GC–MS based non-targeted screening
Source: Brief Bioinform. 2026 Sep 2;27(5):bbag458. doi: 10.1093/bib/bbag458 (PMC13537445; doi:10.1093/bib/bbag458)
Supplement: Supplementary file 1 [file supplementary_material_bbag458.zip › Supplementary_Information_FINAL.docx]

**Supplementary Information**

**Evaluation of Peak Alignment Performance of Commercial and Open-Source Tools for GC×GC-MS Based Non-Targeted Screening**

Andriy Rebryk ^1,2*^, Denice van Herwerden ^3^, Saer Samanipour ^3,4,5^, and Peter Haglund ^1^

*^1^ Department of Chemistry, Chemical Biological Centre (KBC), Umeå University, Linnaeus väg 6, 901 87, Umeå, Sweden*

*^2^* *Amsterdam Institute for Life and Environment (A-LIFE),* *Faculty of Science, Vrije Universiteit Amsterdam, De Boelelaan 1108, 1081 HZ, Amsterdam, The Netherlands*

*^3^ Van 't Hoff Institute for Molecular Sciences, Faculty of Science, University of Amsterdam, Science Park 904, 1098 XH, Amsterdam, The Netherlands*

*^4^ UvA Data Science Center, University of Amsterdam, 1090 GD, Amsterdam, The Netherlands*

*^5^ Queensland Alliance for Environmental Health Sciences (QAEHS), The University of Queensland, Cornwall Street, Woolloongabba, Queensland 4102, Australia*

* *Corresponding author*.

*E-mail address:* [andrew.rebryk@gmail.com](mailto:andrew.rebryk@gmail.com).

**Method S1.** Chromatographic method settings.

**Table S3.** Retention index/retention time data for C10-C36 *n*-alkane mixture in toluene used for retention indexing in this study.

| Name | Absolute R. T. (s)* | Retention Index |
| --- | --- | --- |
| C10 | 613 | 1000 |
| C11 | 803 | 1100 |
| C12 | 1028 | 1200 |
| C13 | 1268 | 1300 |
| C14 | 1508 | 1400 |
| C15 | 1745 | 1500 |
| C16 | 1970 | 1600 |
| C17 | 2188 | 1700 |
| C18 | 2395 | 1800 |
| C19 | 2593 | 1900 |
| C20 | 2780 | 2000 |
| C21 | 2963 | 2100 |
| C22 | 3135 | 2200 |
| C23 | 3303 | 2300 |
| C24 | 3463 | 2400 |
| C25 | 3615 | 2500 |
| C26 | 3765 | 2600 |
| C27 | 3908 | 2700 |
| C28 | 4045 | 2800 |
| C29 | 4178 | 2900 |
| C30 | 4309 | 3000 |
| C31 | 4433 | 3100 |
| C32 | 4555 | 3200 |
| C33 | 4670 | 3300 |
| C34 | 4785 | 3400 |
| C35 | 4898 | 3500 |
| C36 | 5020 | 3600 |

***** Values used in this study were obtained directly from the ChromaTOF peak table of the alkane mixture sample. These values correspond to the first-dimension apex retention times (i.e. the time points of maximum peak intensity) for each detected modulated peak.

**Table S4.** Samples and sample weights used for software evaluation.

| Sample | Sample code | Weight, mg |
| --- | --- | --- |
| Umeå pooled sample | UMPS | 60.9 |
| NORMAN Network European dust trial composite sample | EUCS | 50.6 |
| NIST Standard Reference Material (SRM) 2585 | NIST | 50.7 |
| Procedural blank | BLNK | N/A |

**Workflow S1.** The jAligner4GCxGC workflow.

0. **To use the workflow, several pieces of software must be installed/available**

- LECO ChromaTOF Software for Pegasus BT (version 5.51.50.0)
- Julia (version 1.10.11): open Command Prompt > Type or copy in “juliaup add 1.10.11” > Press “Enter”
- Visual Studio Code notebook: <https://code.visualstudio.com/download>

1. **Library search, classification, and result export in ChromaTOF**

**NB:** The settings for this workflow were chosen based on authors’ experience and do not necessarily reflect default settings of the ChromaTOF software.

a. Open ChromaTOF and create data processing (DP) method for library search

- In “Peak Finding”:
  - Tick off “Enable Peak Finding” and “GC×GC Data”
  - Set “Quantitation Signal” to “TIC”
  - Set “Quantitation mass tolerance” to 0.5 (or any other appropriate value) and select “Da”
  - Set “Integration Baseline” to “Auto-Calculated”
  - Populate “NTD Peak Find Settings” table: set “Start Time” to “Start of Run”, “Report Peaks” to “On”, “Minimum S/N” to “100” (or any other appropriate value), and “Minimum Stick Count” to “4” (or any other appropriate value)

- In “GC×GC Subpeak Combining”, set “Spectral Match Required to combine” to “500” (or any other appropriate value)

- In “Retention Index”, tick off “Enable Retention Index” and select the respective file with retention data (can be prepared by, e.g., acquiring saturated alkane mixture chromatogram in respective solvent; for the RI data used in this work, see Table S3)

- In “Library Search Options”:
  - Tick off “Enable Spectral Similarity Library Search”
  - Set “Maximum Results” to “10” (or any other appropriate value)
  - Set “Minimum mass to library search” to “45” (minimum mass specified in the MS acquisition method)
  - Set “Maximum mass to library search” to “750” (maximum mass specified in the MS acquisition method)
  - Set “Minimum molecular weight allowed” to “0” (or any other appropriate value)
  - Set “Maximum molecular weight allowed” to “1500” (or any other appropriate value)
  - Set “Relative abundance threshold” to “1” (or any other appropriate value)
  - Set “Minimum similarity for matches” to “500” (or any other appropriate value)
  - Set “Minimum similarity before hit is assigned” to “600” (or any other appropriate value)
  - Select the spectral libraries by pressing “Add”
  - Set “Column Phase” to “Semi-Standard Non-Polar” (or any other appropriate value)
  - Tick off “Filter by Retention Index” and set “Tolerance” to “50” (or any other appropriate value)
  - Tick off “Allow estimated RI substitution for non-polar types” to enable estimated RI assignment in case Semi-Standard Non-Polar RI is missing for a given compound
  - Tick off “Use other non-polar column type data if selected non-polar type data is absent” to enable assignment in case the data for the used column type is missing

- Save and name method by pressing “Save as”

b. Create DP method for feature classification

- In “Classification”:
  - Tick off “Enable Classification”
  - Add class data in “Classes to use to classify the peaks” by pressing “Add” and navigating to the respective file.

- The classification file is created using “Create Regions” option in “Classifications” tab. Then, classes are created using “Create New Class” option and regions are assigned to classes using “Assign Region To Class” functionality in “Classifications” tab.

- Additionally, spectral peak filters are added to classes as follows: “Classification – “Class file name” tab 🡪 Press on any class name in “Class” column 🡪 Choose filter from a drop-down menu 🡪 Press “OK”.

- Peak filters are created as follows: “Peak Table – Sample name” tab 🡪 Press on “Filter” (filtration funnel icon) 🡪 Choose “Spectral” tab 🡪 Create spectral peak filter using statements 🡪 Save and name the filter by pressing “Save Filter Set” 🡪 Press “OK”.

- For more information about regions and filters, see “Classifications Overview” and “Working with Peak Filters – Spectral” sections and any other related section of the ChromaTOF manual.
- Save and name DP classification method by pressing “Save as”

c. Go to “Acquired Samples” and select all sample files to be processed. To begin DP, right mouse click on the selected samples 🡪 Data Processing. The new window will be called to the sample files location where the samples to be processed are highlighted; any additional samples can be added to the DP list at this point. Press “Select” 🡪 Select DP method, e.g., library search or classification 🡪 Press “Select”
d. Create data export method

- In “Peak Data ASCII Export Options”:
  - Tick off “Export peak information in ASCII CSV format” and specify export directory
  - Set “Field Separator” to “Tab”
  - Tick off “Add Not Found Analytes”
- In “Columns”, select which information columns to be exported by highlighting them and pressing “Add >>”. For the next steps of the workflow, the following data columns should be included: “Name”, “R.T. (s)”, “Retention Index”, “Lib. RI”, Synonyms”, “Synonyms List”, “Observed Ion m/z”, “Expected Ion m/z”, “Formula”, “CAS”, “InChIKey”, “Actual Masses”, “Base Mass”, Quant Masses”, “FWHH (s)”, “Probability” “Similarity”, “Reverse”, “Area”, “Height”, “Peak S/N”, “ “Quant S/N”, “Conc. Unit”, “Classifications”, “Spectrum”
  - In “Spectra”, tick off “Export the peak true spectrum as part of the information for each peak.” and set “Format of Intensity values” to “Relative”
- Save and name method by pressing “Save as”

e. Go to “Acquired Samples” and select all sample files to be exported. To begin export, right mouse click on the highlighted samples 🡪 Data Processing. The new window will be called to the sample files location where the samples to be exported are highlighted; any additional samples can be added to the list at this point. Press “Next” 🡪 Select DP file export method 🡪 Press “OK”
2. **Sample preprocessing, inter-sample peak alignment, and postprocessing in jAligner4GCxGC**
   a. Create a working folder for input files and alignment result files and copy only exported LECO TXT files that should be aligned to the working folder
   b. Follow the steps specified in the jAligner4GCxGC manual (available at <https://github.com/r3bryk/jAligner4GCxGC>) to install necessary applications/tools
   c. Open run_me.jl file in Visual Studio Code notebook
   d. Enable using packages by highlighting “using Pkg” and pressing “Ctrl + Enter”
   e. Get the package by highlighting “Pkg.add(url="https://github.com/r3bryk/jAligner4GCxGC")” and pressing “Ctrl + Enter”. This procedure will take some time. When done, you will see a message “julia>” in the terminal field; this message will appear after any operation is completed and Julia is ready to proceed
   f. Load/import the package for use by highlighting “using jAligner4GCxGC” and pressing “Ctrl + Enter”
   g. Specify the path to input files as “path2files = "C:\\Users\\CountDracula\\Alignment””, where "C:\\Users\\CountDracula\\Alignment” is the path to the working folder. Load input files by highlighting the line and pressing “Ctrl + Enter”
   h. Specify the parameters for the preprocessing functions that will be used (see the manual for more information) and execute the functions by highlighting them and pressing “Ctrl + Enter”
   i. Specify alignment function “feature_align_external” parameters, for example:

- h_thresh = 10000000
- rt_tol_lowA = 11
- rt_tol_highA = 11
- rt2_tol_lowA = 0.1
- rt2_tol_highA = 0.1
- sim_thresh = 0.8
- mz_tol = 0.025
- overviewMerging = "height"
- weights = [5, 1, 1]
- tr1_parts = 5
- numfrags = 150
- similarity_method = "DISCO"

The parameters were chosen based on the authors’ experience. Various parameter combinations were evaluated with the aim to get the higher alignment accuracy, the lower number of replicate features, the lowest number of misalignments, and avoiding merging of closely eluting compounds with similar spectra such as terpenes.

j. Align samples by highlighting the line with the alignment function “feature_align_external” and pressing “Ctrl + Enter”
k. Specify the parameters for the postprocessing functions that will be used (see the manual for more information) and execute the functions by highlighting them and pressing “Ctrl + Enter”
l. Output CSV files, including the main alignment result table file “YYMMDD_Aligned”, will be created in the working folder.

**Workflow S2.** The Guineu workflow.

0. **To use the workflow, several pieces of software must be installed/available**

- LECO ChromaTOF Software for Pegasus BT (version 5.51.50.0)
- Guineu (version 1.0.3)
- Python 3 (version 3.13.5): <https://www.python.org/downloads>
- Visual Studio Code notebook: <https://code.visualstudio.com/download>

1. **Library search, classification, and result export in ChromaTOF**

**NB:** The settings for this workflow were chosen based on authors’ experience and do not necessarily reflect default settings of the ChromaTOF software.

a. Open ChromaTOF and create data processing (DP) method for library search

- In “Peak Finding”:
  - Tick off “Enable Peak Finding” and “GC×GC Data”
  - Set “Quantitation Signal” to “TIC”
  - Set “Quantitation mass tolerance” to 0.5 (or any other appropriate value) and select “Da”
  - Set “Integration Baseline” to “Auto-Calculated”
  - Populate “NTD Peak Find Settings” table: set “Start Time” to “Start of Run”, “Report Peaks” to “On”, “Minimum S/N” to “100” (or any other appropriate value), and “Minimum Stick Count” to “4” (or any other appropriate value)

- In “GC×GC Subpeak Combining”, set “Spectral Match Required to combine” to “500” (or any other appropriate value)

- In “Retention Index”, tick off “Enable Retention Index” and select the respective file with retention data (can be prepared by, e.g., acquiring saturated alkane mixture chromatogram in respective solvent; for the RI data used in this work, see Table S3)

- In “Library Search Options”:
  - Tick off “Enable Spectral Similarity Library Search”
  - Set “Maximum Results” to “10” (or any other appropriate value)
  - Set “Minimum mass to library search” to “45” (minimum mass specified in the MS acquisition method)
  - Set “Maximum mass to library search” to “750” (maximum mass specified in the MS acquisition method)
  - Set “Minimum molecular weight allowed” to “0” (or any other appropriate value)
  - Set “Maximum molecular weight allowed” to “1500” (or any other appropriate value)
  - Set “Relative abundance threshold” to “1” (or any other appropriate value)
  - Set “Minimum similarity for matches” to “500” (or any other appropriate value)
  - Set “Minimum similarity before hit is assigned” to “600” (or any other appropriate value)
  - Select the spectral libraries by pressing “Add”
  - Set “Column Phase” to “Semi-Standard Non-Polar” (or any other appropriate value)
  - Tick off “Filter by Retention Index” and set “Tolerance” to “50” (or any other appropriate value)
  - Tick off “Allow estimated RI substitution for non-polar types” to enable estimated RI assignment in case Semi-Standard Non-Polar RI is missing for a given compound
  - Tick off “Use other non-polar column type data if selected non-polar type data is absent” to enable assignment in case the data for the used column type is missing

- Save and name method by pressing “Save as”

b. Create DP method for feature classification

- In “Classification”:
  - Tick off “Enable Classification”
  - Add class data in “Classes to use to classify the peaks” by pressing “Add” and navigating to the respective file.

- The classification file is created using “Create Regions” option in “Classifications” tab. Then, classes are created using “Create New Class” option and regions are assigned to classes using “Assign Region To Class” functionality in “Classifications” tab.

- Additionally, spectral peak filters are added to classes as follows: “Classification – “Class file name” tab 🡪 Press on any class name in “Class” column 🡪 Choose filter from a drop-down menu 🡪 Press “OK”.

- Peak filters are created as follows: “Peak Table – Sample name” tab 🡪 Press on “Filter” (filtration funnel icon) 🡪 Choose “Spectral” tab 🡪 Create spectral peak filter using statements 🡪 Save and name the filter by pressing “Save Filter Set” 🡪 Press “OK”.

- For more information about regions and filters, see “Classifications Overview” and “Working with Peak Filters – Spectral” sections and any other related section of the ChromaTOF manual.
- Save and name DP classification method by pressing “Save as”

c. Go to “Acquired Samples” and select all sample files to be processed. To begin DP, right mouse click on the selected samples 🡪 Data Processing. The new window will be called to the sample files location where the samples to be processed are highlighted; any additional samples can be added to the DP list at this point. Press “Select” 🡪 Select DP method, e.g., library search or classification 🡪 Press “Select”
d. Create data export method

- In “Peak Data ASCII Export Options”:
  - Tick off “Export peak information in ASCII CSV format” and specify export directory
  - Set “Field Separator” to “Tab”
  - Tick off “Add Not Found Analytes”
- In “Columns”, select which information columns to be exported by highlighting them and pressing “Add >>”. For step 2 of the workflow, the following data columns should be included: “Name”, “Synonyms List”, “Formula”, “CAS”, “InChIKey”, “R.T. (s)”, “Retention Index”, “Lib. RI”, “Actual Masses”, “Base Mass”, “Expected Ion m/z”, “Similarity”, “Area”, “Peak S/N”, “Quant Masses”, “Quant S/N”, “Conc. Unit”, “Classifications”, “Type”, “Group”, “Spectrum”. In case other data columns were exported, the Python 3 script from step 2 has to be adjusted accordingly
  - In “Spectra”, tick off “Export the peak true spectrum as part of the information for each peak.” and set “Format of Intensity values” to “Relative”
- Save and name method by pressing “Save as”

e. Go to “Acquired Samples” and select all sample files to be exported. To begin export, right mouse click on the highlighted samples 🡪 Data Processing. The new window will be called to the sample files location where the samples to be exported are highlighted; any additional samples can be added to the list at this point. Press “Next” 🡪 Select DP file export method 🡪 Press “OK”
2. **Low-resolution (unit resolution) spectral conversion using the HiRes2LowRes_Converter.py**
   a. To be aligned in Guineu software, which can handle only unit-resolution MS data, the exported text files with high-resolution MS data have to be converted to low-resolution MS data (unit resolution). It is done by the HiRes2LowRes_Converter.py Python 3 script (available at <https://github.com/r3bryk/HiRes2LowRes_Converter>). Also, a number of other important amends are made by the script (see below). All the amends are made in a way to fit the file format accepted by Guineu (v. 1.0.3). The input data should be in the format of tab-separated TXT files (.txt) and at least the following data (columns) have to be included:

“Name” “R.T. (s)” “Similarity” “Area” “Spectrum” “Classifications”

**NB:** Guineu takes one “R.T. (s)” column that contains 1st dimension RT (RT1) and 2nd dimension RT (RT2) values stored as “RT1, RT2”.

b. To use HiRes2LowRes_Converter.py do the following:
- Open HiRes2LowRes_Converter.py file in Visual Studio Code notebook and do: right mouse click anywhere in the notebook 🡪 Run Python 🡪 Run Python File in Terminal. Alternatively, press the play icon in the top right corner. The new window will be called.
- Navigate to the folder where the files to be processed are stored, select the files and press “Open”. The processed files will be saved to the same location under the name “YYMMDD_LR_InitialFilename”, where “YYMMDD” is today’s date and “LR” means “Low Resolution”. In case the initial filename already contains date in a format "YYMMDD", it will be deleted, and today’s date will be added to the output filename instead
  c. HiRes2LowRes_Converter.py takes tab-separated TXT files and does the following:
  - Checks whether “R.T. (s)” column is present in the input file and picks the right algorithm for further processing. Otherwise, the script returns error
  - Deletes features (rows) for which there are empty cells in “Area” column
  - Removes all features (rows), for which there is a value “Bleed”, “Toluene”, or “DCM” in “Classifications” column. The classification is done in ChromaTOF using spectral filter and region functionalities
  - Removes all features (rows) where "Name" column contains unwanted keywords, e.g., “silo” for any “siloxane” or “syloxy” compounds or “bora” for any “borane” or “borate” features
  - Removes all features (rows) where "Name" column contains unwanted keywords and does not contain specific m/z, e.g., compounds with spectra similar to toluene (m/z 91 or 92) if their spectra do not contain other higher m/z values, like molecular ions
  - Rounds 1^st^ dimension RT (RT1) values of the column “R.T. (s)”
  - Renames “non-hit” entries (unknown features; no match in the NIST library) from “Peak #” to “Peak_RT1 (rounded)_RT2”
  - Rounds “Base Mass” column values
  - Populates empty “Similarity” column cells for “non-hits” with a value of “800” to enable simultaneous processing and alignment of both “hits” and “non-hits”; otherwise, “non-hits” are skipped during the inter-sample peak alignment in Guineu (“Score Alignment” feature)
  - Rounds “Spectrum” column values (*m/z* & intensity) to unit resolution (the nearest integer using banker’s rounding (“round half to even”) approach: values <*.5 round down (e.g., 44.15 and 44.49 to 44), values >*.5 round up (e.g., 44.51 and 44.90 to 45), values *.5 round to even (e.g., 44.50 to 44 and 45.50 to 46), and values *.00 stay the same (e.g., 45.00 to 45)). In case duplicate *m/z* 's appear in a given spectrum after rounding, only one *m/z* is stored and the respective intensity values are added; otherwise, one intensity value is assigned to one *m/z* value

**NB:** HiRes2LowRes_Converter.py crashes if:

- an empty file is submitted
- a file does not contain “R.T. (s)” column and/or any other necessary column
- data submitted was already converted
- for undiscovered (yet) reasons

3. **Inter-sample peak alignment in Guineu**

**NB:** The settings for this workflow were chosen based on authors’ experience and do not necessarily reflect default settings of the Guineu software.

a. Open Guineu and import the converted files: File 🡪 Open GC×GC-Tof File 🡪 Specify Separator: \t 🡪 Press “…” 🡪 Choose files and press “Select file” 🡪 Press “OK”
b. To align the samples, click on each sample in the “Data set Files” panel to select them, then do the following: Click on “GC×GC-MS” tab 🡪 Score Alignment 🡪 Specify alignment parameters, for example:
   - RT Lax: 11
   - RT2 Lax: 0.1
   - RI Lax: 10
   - RT penalty: 5
   - RT2 penalty: 30
   - RTI penalty: 30
   - Minimum Spectrum Match: 0.8
   - Bonus for matching names: 0
   - Drop peaks with similarity less than: 600
   - For more information on the settings, check “Help” tab and/or the paper [1]
   - Press “OK”

The parameters were chosen based on the authors’ experience. Various parameter combinations were evaluated with the aim to get the higher alignment accuracy, the lower number of replicate features, the lowest number of misalignments, and avoiding merging of closely eluting compounds with similar spectra such as terpenes.

c. To save the alignment table, right mouse click on the alignment name in the left panel 🡪 Save Dataset in a File 🡪 Press “…” 🡪 Choose folder and type file name and press “Select file” 🡪 Choose file format in GCGC type, e.g., csv 🡪 Select column to export in Export GCGC elements 🡪 Press “OK”.

**Workflow S3.** The ChromaTOF Sync 2D workflow.

0. **To use the workflow, several pieces of software must be installed/available**

- LECO ChromaTOF Sync 2D (version 2.0.15.0)
- Python 3 (version 3.13.5): <https://www.python.org/downloads>
- Visual Studio Code notebook: <https://code.visualstudio.com/download>

1. **Inter-sample peak alignment in ChromaTOF Sync 2D**

**NB:** The settings for this workflow were chosen based on authors’ experience and do not necessarily reflect default settings of the ChromaTOF Sync 2D software.

a. Open ChromaTOF Sync 2D and press “Begin new alignment”

- In “Alignment name”, specify resulting file name
- In “Alignment type”, pick “GCxGC”
- In “Sample set”:
  - Choose desired database by double-clicking on its name
  - Choose desired sample folder by double-clicking on its name
  - Choose samples to be aligned by highlighting them and dragging to “Samples to compare” pane

- In “Alignment method”:
  - In “Alignment”, specify alignment parameters, for example:
    - Expected FWHH D2 (sec): 0.05
    - Signal to noise: 10
    - Expected maximum D1 retention shift (sec): 11
    - Expected maximum D2 retention shift (sec): 0.1
    - Any other parameters if needed (not specified in this study)
  - In “Library search”, specify library search parameters, for example:
    - Library Identity Search Mode: Normal
    - Library Search Mode: Forward
    - Rank hits by: Probability
    - Maximum number of hits: 10
    - Minimum mass to library search: 45
    - Maximum mass to library search: 750
    - Minimum molecular weight allowed: 0
    - Maximum molecular weight allowed: 1500
    - Relative abundance threshold (0-998): 1
    - Minimum similarity for matches (0-999): 500
    - Minimum similarity before hit is assigned (0-999): 600
    - Tick off “Retrieve retention indices”: Semi-standard non-polar
    - Tick off “Filter hits that are at least 50 units out of tolerance with computed retention index”
    - Tick off desired “Libraries to search”: mainlib, replib

- In “Landmarks”, specify name, retention times (R.T. (sec)), and retention indices (R.I.) for compounds that will be used for retention indexing

- Press “Begin GCxGC alignment”

2. **External postprocessing for ChromaTOF Sync 2D alignment data**
a. Because of the nature of the alignment algorithm that ChromaTOF Sync 2D uses, all the features are usually “found” in all the samples, however, with very small areas, e.g., 770, instead of 0. This creates problems when it comes to detection frequency (DF) calculation, which was needed in this study for alignment performance evaluation

b. Python script (used in this study) or another software for plotting can be used to distinguish between real peaks and peaks potentially with area values of 0. Histogram plot for distribution of log10-transformed peak areas can be created and antilog of the intersect of the two clusters (~4), giving an area cutoff of antilog10(4) = 10^4^ = 10000. Then, all the area values under 10000 are replaced with 0

c. In addition, machine learning (ML)-based modeling methods, such as Gaussian mixture model (GMM) and/or kernel density estimation (KDE), can be used for area cutoff estimation. However, with this rather small dataset, they do not give reliable results

**Table S5A**. jAligner4GCxGC performance speed evaluation.

| Step | Time spent** |
| --- | --- |
| File conversion, pre-alignment data filtration* | 0 h 2 m 17 s |
| Peak alignment (10480 features)* | 0 h 1 m 31 s |
| Post-alignment data filtration & feature merging* | 0 h 0 m 33 s |
| PubChem data retrieval (294 identified features) | 0 h 9 m 29 s |
| Additional library searching, NIST2023 (10011 features) | 11 h 00 m 37 s |
| Library searching visualization (10011 features / 30033 graphs) | 0 h 44 m 34 s |
| *Alignment activities time (10480 features) | **0 h 4 m 21 s** |
| Total time spent | **12 h 23 m 51 s** |

** Time measurements come from the internal logs (printing statements) reported by the software.

**Table S5B**. ChromaTOF data pre-processing speed evaluation.

| **Step*** | **Time spent**** |
| --- | --- |
| Peak finding | 0 h 17 m 21 s |
| Area & height calculation | 0 h 0 m 15 s |
| Library searching, NIST2023 | 0 h 3 m 18 s |
| **Total time spent** | **0 h 20 m 54 s** |

* The steps also include GC×GC subpeak combining, retention indexing, and classification; however, those steps are not explicitly stated in the software log file.

** Indicative times for a single sample.

**Table S6**. ChromaTOF Sync 2D performance speed evaluation.

| Step | Time spent** |
| --- | --- |
| Peak finding | 2 h 35 m 42 s |
| Peak alignment (5069 features)* | 0 h 4 m 22 s |
| Finalization, quant profile calculation, signal-to-noise filtration, and RI calculation* | 0 h 4 m 28 s |
| Library searching, NIST2023 (5069 features) | 0 h 7 m 46 s |
| Validation | 0 h 1 m 2 s |
| *Alignment activities time (5069 features) | **0 h 8 m 50 s** |
| Total time spent | **2 h 53 m 20 s** |

** Time measurements come from the alignment log file generated by the software.

**Table S7**. Specifications of the device used in this study.

| Processor | AMD Ryzen Threadripper PRO 5955WX 16-Cores |
| --- | --- |
| Processor speed | 4.00 GHz |
| Installed RAM | 128 GB usable (4 slots × 32 GB), 3200 MT/s, DDR4 ECC Registered (RDIMM) |
| System storage (software) | Micron 3400 NVMe, 2 TB NVMe SSD, ~6600 MB/s |
| System storage (files) | HGST Ultrastar He10, 8 TB SATA HDD, 7200 RPM, ~250 MB/s |
| System type | 64-bit operating system, ×64-based processor |
| Edition | Windows 11 Enterprise, v. 24H2, build 26100.3194 |
| GPU | NVIDIA T400 |
| Dedicated GPU memory | 4 GB |
| Shared GPU memory | 64 GB |

**Table S8**. Information about closely eluting terpenes used in this study.

| Name | Library RI | Formula | Base mass | M.w. | CAS# |
| --- | --- | --- | --- | --- | --- |
| *α*-Pinene | 937 | C10H16 | 93 | 136 | 80-56-8 |
| Camphene | 952 | C10H16 | 93 | 136 | 79-92-5 |
| Sabinene | 974 | C10H16 | 93 | 136 | 3387-41-5 |
| Aniline | 977 | C6H7N | 93 | 93 | 62-53-3 |
| (-)-*β*-Pinene | 978 | C10H16 | 93 | 136 | 18172-67-3 |
| *β*-Myrcene | 991 | C10H16 | 93 | 136 | 123-35-3 |
| *α*-Phellandrene | 1005 | C10H16 | 93 | 136 | 99-83-2 |
| 3-Carene | 1011 | C10H16 | 93 | 136 | 13466-78-9 |
| *γ*-Terpinene | 1060 | C10H16 | 93 | 136 | 99-85-4 |
| Terpinolene | 1088 | C10H16 | 93 | 136 | 586-62-9 |

**Figure S1**. Chromatographic region with closely eluting compounds whose spectra contain mass 93 derived from the reference standard mixture sample (log-scaled). Peak A corresponds to aniline, peak B corresponds to sabinene, and peak C corresponds to (-)-*β*-pinene.

**Figure S2**. Spectra of sabinene, (-)-*β*-pinene, and aniline (reproduced from the NIST 2023 EI mass spectral library using MS Search software).

**Sabinene**

**(-)-*β*-Pinene**

**Aniline**

**References**

1. Castillo, S., Mattila, I., Miettinen, J., Oresic, M. and Hyötyläinen, T., 2011. Data analysis tool for comprehensive two-dimensional gas chromatography/time-of-flight mass spectrometry. Analytical Chemistry, 83(8), pp.3058-3067. <https://doi.org/10.1021/ac103308x>
